# *Cis*-regulatory elements in conserved non-coding sequences of nuclear receptor genes indicate for crosstalk between endocrine systems

**DOI:** 10.1515/med-2021-0264

**Published:** 2021-04-12

**Authors:** Maria Araceli Diaz Cruz, Dan Lund, Ferenc Szekeres, Sandra Karlsson, Maria Faresjö, Dennis Larsson

**Affiliations:** Research School of Health and Welfare, School of Health and Welfare, Jönköping University, Jönköping, Sweden; Department of Natural Science and Biomedicine, School of Health and Welfare, Jönköping University, Jönköping, Sweden; Department of Biomedicine, School of Health Sciences, University of Skövde, Skövde, Sweden; Sahlgrenska University Hospital, Gothia Forum for Clinical Research, Gothenburg, Sweden

**Keywords:** conserved sequences, transcription factor binding sites, splicing sites, nuclear receptor binding domains, crosstalk

## Abstract

Nuclear receptors (NRs) are ligand-activated transcription factors that regulate gene expression when bound to specific DNA sequences. Crosstalk between steroid NR systems has been studied for understanding the development of hormone-driven cancers but not to an extent at a genetic level. This study aimed to investigate crosstalk between steroid NRs in conserved intron and exon sequences, with a focus on steroid NRs involved in prostate cancer etiology. For this purpose, we evaluated conserved intron and exon sequences among all 49 members of the NR Superfamily (NRS) and their relevance as regulatory sequences and NR-binding sequences. Sequence conservation was found to be higher in the first intron (35%), when compared with downstream introns. Seventy-nine percent of the conserved regions in the NRS contained putative transcription factor binding sites (TFBS) and a large fraction of these sequences contained splicing sites (SS). Analysis of transcription factors binding to putative intronic and exonic TFBS revealed that 5 and 16%, respectively, were NRs. The present study suggests crosstalk between steroid NRs, e.g., vitamin D, estrogen, progesterone, and retinoic acid endocrine systems, through *cis*-regulatory elements in conserved sequences of introns and exons. This investigation gives evidence for crosstalk between steroid hormones and contributes to novel targets for steroid NR regulation.

## 1 Introduction

The nuclear receptor superfamily (NRS) is composed of ligand-activated transcription factors that control important developmental and physiological processes, by regulating gene expression when bound to specific DNA sequences. The NRS is crucial for regulating cell, organ, and body homeostasis, and an alteration of expression of specific nuclear receptors (NRs) is one of the causes of many human diseases [1,2,3].

Introns are a major proportion of DNA in both the plant and mammalian genome and are considered relevant components for genome adaptation [4]. They are not simply removed after RNA processing and are responsible for chromatin modification, transcription, RNA splicing, editing, translation, and gene expression [4,5,6,7]. The presence of introns elevates gene expression in a wide range of organisms including mammals [6,8,9]. Interestingly, intronic DNA sequences act as internal promoters that can be more important than the proximal promoter and constitute unrecognized binding sites for genes transcribed by the RNA polymerase II [6,9]. Even in the absence of the promoter in some genes, mRNA accumulation can be stimulated by the presence of certain regulatory intronic sequences [8].

Several reports have suggested a positive association between the complexity of an organism and the intronic fraction in the genome, which seems to be responsible for species-specific adaptations [7,10]. Integrated genomic analysis suggests that non-coding sequences in conserved sites usually are enriched with regulatory binding sites [11]. The same high abundance of regulatory binding sites has not been observed in exons [12]. This, therefore, suggests that the prevalence of conserved non-coding sequences (CNSs) among species is related to the preservation of a specific function and/or gene regulation and RNA splicing [5,13].

The regulatory elements located in non-coding intron and exon DNA sequences (*cis*-regulatory elements, CREs) are often transcription factor binding sites (TFBS) or splicing factor binding sites (SFBS) [14]. Mutations in CREs result in a significant reduction in target gene transcription and predispose individuals to a wide variety of disorders such as diabetes and cancer [15,16,17]. One example of a disease caused by this alteration is prostate cancer, where the androgen receptor (AR) is overexpressed in most castration-resistant patients [18]. Ethnicity and geography are risk factors for prostate cancer [19] as well as inherited mutations of the Breast cancer type 1 susceptibility protein (*BRCA1*) and Breast cancer type 2 susceptibility protein (*BRCA2*) genes, which are connected to estrogen regulation [20]. Ethnicity and geography (high latitudes) are associated with, e.g., low serum levels of vitamin D, which leads to decreased anti-inflammatory effects as well as decreased apoptotic activity and an increased proliferation [21]. The *BRCA1* gene has been reported to be a co-regulator of the AR [22] and inhibits estrogen receptor α (ERα) signaling [23]. However, the interaction between the *BRCA2* gene and estrogen signaling is indirect, and mutations in the *BRCA2* are associated with decreased activity of gene DNA recombination and repair processes [24].

In prostate cancer, there is an imbalance in the crosstalk between different steroid hormones (e.g., androgens, estrogens, glucocorticoids, progesterone, retinoids, and vitamin D) leading to uncontrolled cell growth. Crosstalk between steroid endocrine systems, on a genetic level as well as in signaling pathways, has been reported but not fully understood [25]. Deepening our knowledge on the crosstalk between steroid endocrine systems, through their NRs, is of importance to understand the initiation and progression of hormone-driven cancers [25]. Little is known about NR interaction with non-coding conserved nucleic acid sequences. An understanding of the conserved intronic region distribution across species will enable the identification of candidate regulatory sequences. These sequences could interact with NRs evoking a change in the NR DNA binding and the NRs regulation of expression/repression of specific genes.

Our hypothesis is that steroid endocrine systems regulate gene expression through interactions with introns and exons. This study thus aimed to investigate crosstalk between steroid NRs in conserved intron and exon sequences, with a focus on steroid NRs involved in prostate cancer etiology. For this purpose, conserved intronic and exonic NRS sequences were analyzed with a focus on *cis*-regulatory elements (CREs) and their involvement in fundamental processes such as growth, differentiation, homeostasis, development, and metabolism.

## 2 Material and methods

Intron and exon sequences from the 49 genes of the NRS, translated from pre-mRNA transcripts to the corresponding DNA sequence, were retrieved from the Ensembl genome database (Table S1) [26]. The transcripts selected were orthologous, having the same position and phase relative to the coding sequence, from five different mammalian species: *Homo sapiens*, *Gorilla gorilla*, *Pan troglodytes*, *Mus musculus*, and *Rattus norvergicus* (Table S1). These specific mammalian species were selected based on their close phylogenetic relationship, which implies high sequence conservation probability, and their usage for modeling species to humans [13,27,28]. Since some conserved genomic regions in primates and rodents recently have been identified as unique and responsible for new emerging functions, species from both orders were included in this analysis [13].

### 2.1 Basic conserved sequence detection method (BCSDm)

The Basic Conserved Sequence Detection method (BCSDm) extracts the most conserved sequences (without insertions or deletions) and their location within the specific sequence region.

The BCSDm implemented in Python 3.6 using Biopython [29] is based on the combination of three methods: (1) alignment of the different sequences, (2) extraction of the alignment profile and its position score matrix (PSSM), and (3) obtainment of the conserved nucleotide patterns and their position in the alignment (Figure S1).

The alignment was performed for each gene sequence between the five mammalian species using the multiple alignment program MAFFT [30]. From the resulting alignment file, an alignment profile summarizing the alignment for the five species was obtained. The dumb consensus method was selected for extracting the alignment profile and for calculating the number of each nucleotide type at each position of the alignment for all the sequences [29]. If the percentage of the most common nucleotide type was greater than the default threshold (0.7), the nucleotide was added to the alignment profile. This method was used to avoid gaps in the extracted sequence pattern. After obtaining the alignment profile, a PSSM was calculated to represent the probabilities of the occurrence of each nucleotide in the consensus sequence. The conserved patterns were extracted from the PSSM by the following conditions. First, each position selected from the score matrix should be 100% conserved, to avoid gaps, insertions or substitutions. Second, to include TFBS and to exclude random appearance in the selection of conserved patterns, a threshold of ≥15 consecutive nucleotides was applied.

All the 49 genes from the NRS were analyzed with BCSDm. Of these, 25 genes had at least one conserved intronic pattern and were thus further analyzed. A total of 1,044 conserved intron patterns in these 25 genes were extracted using BCSDm. Conserved sequences were grouped according to their ordinal position in the transcript (called intron 1 to intron 11). The percentage of conserved sequences was calculated for each intron group. To avoid an unequal number of introns between NR genes, the conserved patterns obtained were normalized to the number of genes containing each intron. Moreover, the number of conserved patterns in each intron was normalized to their sequence length.

Exon sequences, from the same 25 NRS genes described earlier, were also analyzed with BCSDm. A total of 552 conserved exon patterns in these 25 genes were extracted and further analyzed.

### 2.2 Analysis of CREs

Each conserved intronic and exonic pattern in the 25 selected NRS genes was extracted by BCSDm and scanned for putative binding sites for transcription factors [31] in the CIS-BP Database [32]. The search was performed using the species parameter *Homo sapiens* since the aim was to find out the relevance of these patterns only for the human species. The motif model was set to the standard scoring system option which is position weight matrices (PWMs) – log-odds [33]. To be more restrictive in allowing mutations and to increase the likelihood of the TFBS predicted, the log-odds threshold was set to ten. To remove sparse sequences, only matching sequences with ≥10 consecutive nucleotides were considered potential TFBS.

After identification of potential TFBS in conserved sequences, the transcription factor binding domain (TFBD) family of the transcription factors inferred to bind these TFBS sequences was analyzed. A classification depending on the domain family type was carried out for each TFBDs. Thirty-seven different family domain types were identified and the ten with the highest number of TFBS were selected for further analysis of the introns. A small fraction of all analyzed TFBDs were NR binding domains. TFBS identified as NR binding sites were further analyzed and compared between introns and exons.

The conserved intronic patterns were further analyzed using the Human Splicing Finder (HSF) database to determine whether SS motifs were contained in their sequences [34]. This tool enables the prediction of potential donor and acceptor sites for the sequence introduced. The analysis used the default prediction algorithms (HSF and MaxEnt). The consensus value (HSF) was increased from 65 to 75 to allow higher similarity and confidence of a true splice site to be obtained [34,35]. Thus, the sequences with a consensus value of ≥75 (HSF) and ≥3 (MaxEnt) were classified as containing a splice site.

The conserved intronic patterns were classified as TFBS or SS depending on their content in regulatory elements. This classification revealed that some patterns contained exclusively TFBS or SS and/or both TFBS and SS in the same sequence. Four groups were derived from these results: TFBS, TFBS-SS, SS, and not identified.

The number of TFBS and SS in non-conserved sequences from the same gene intronic regions as the conserved sequences were used as controls. These sequences were scanned into the CIS-BP Database for TFBS hits and with HSF for splicing signals, with the same parameters as for the conserved sequences analysis. Moreover, a classification of TFBD families for TFBS in non-conserved sequences was used as a control. To randomly obtain the non-conserved sequences (*n* = 1,044), the BCSDm program was modified to extract nucleotides from the PSSM that were less than 100% conserved while maintaining a threshold of ≥15 consecutive nucleotides to generate a sequence.

### 2.3 Statistical analysis

The distribution of conserved sequences among different introns was assessed by the Mann–Whitney *U* test. The frequency distribution of the four groups (TFBS, TFBS-SS, SS, and not identified) among introns was analyzed using the Chi-square test. The number of TFBS as well as SS in conserved and non-conserved sequences was analyzed by the Wilcoxon paired non-parametric test. The number of TFBS for each TFBD family, between conserved and non-conserved patterns, was assessed by the Chi-square test. All the statistical analyses were performed in GraphPad Prism version 7.04 (GraphPad Software, La Jolla, California, USA). Statistically significant differences were set to ns: Not significant, *P < 0.05, **P < 0.01, ***P < 0.001 and ****P < 0.0001.


**Ethical approval:** The conducted research is not related to either human or animal use.

## 3 Results

**Figure 1:**
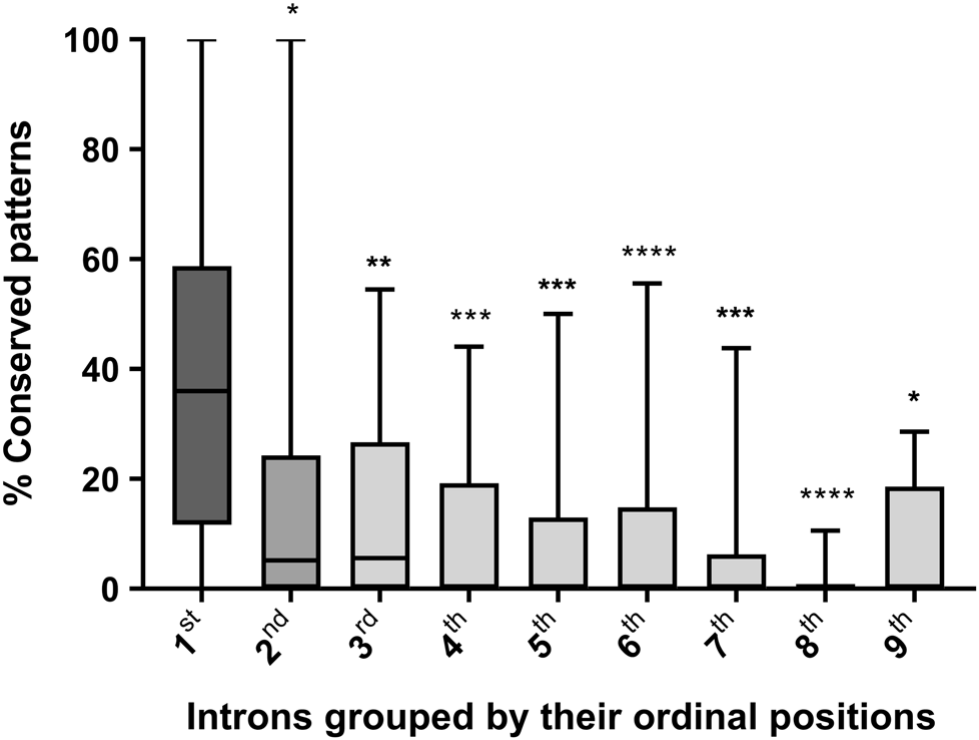
Percentage of sequence conservation for each intron group (1st to 9th) from 25 genes generated from the NRS analysis. Mann–Whitney *U* statistical test was performed to assess the differences of conserved sequences between different groups of introns (*P < 0.05, **P < 0.01, ***P < 0.001, ****P < 0.0001).

Out of 25 NRS genes analyzed, the first intron in each gene (intron 1) had significantly higher sequence conservation (35%) when compared with downstream introns (introns 2–9, Figure 1). This was confirmed even after normalization for the number of genes and the sequence length (Tables S2 and S3, respectively). The number of conserved patterns generally tended to decrease with an ordinal position of the intron.

Seventy-nine percent of the conserved patterns showed putative TFBS in the CIS-BP Database for *Homo sapiens*. In the case of the distribution of TFBS per intron, the frequency was preserved in the range from 70 to 89%, except for intron 8 that was 38% (Table 1). The non-conserved sequences showed that 69% of putative TFBS were overlapping (Table S4). Overlapping TFBS within the conserved sequences was 79%. Hence, 10% of the TFBS sequences, detected among the conserved patterns, are associated with conservation. Thus, the number of TFBS in conserved sequences was significantly higher when compared with non-conserved sequences (*P* < 0.05).

**Table 1:** Frequency distribution of the conserved patterns identified as TFBS for the introns 1–9

Intron	1	2	3	4	5	6	7	8	9	Total
Cons patterns	385	147	173	88	121	84	20	8	18	1,044
TFBS	294	121	150	67	96	63	14	3	16	824
SS	137	41	58	28	42	30	6	4	5	351
TFBS/Cons patterns (%)	76	82	87	76	79	75	70	38	89	
SS/Cons Patterns (%)	36	28	34	32	35	36	30	50	28	

*Cons patterns, conserved patterns; TFBS, Transcription factor binding sites identified in the conserved patterns; SS, Splicing sites identified in the conserved patterns; % TFBS/Cons patterns, percentage of TFBS with respect to the total number of conserved patterns; % SS/Cons patterns, percentage of SS with respect to the total number of conserved patterns.

SS was found in 33% of the conserved patterns of *Homo sapiens* in the HSF Database (Table 1). The non-conserved sequences contained 23% SS (Table S5) and 33% of the conserved sequences overlapped with SS. Hence, 10% of these SS are associated with conserved sequences. The number of SS in conserved patterns was significantly higher when compared with non-conserved patterns (*P* < 0.01).

**Figure 2:**
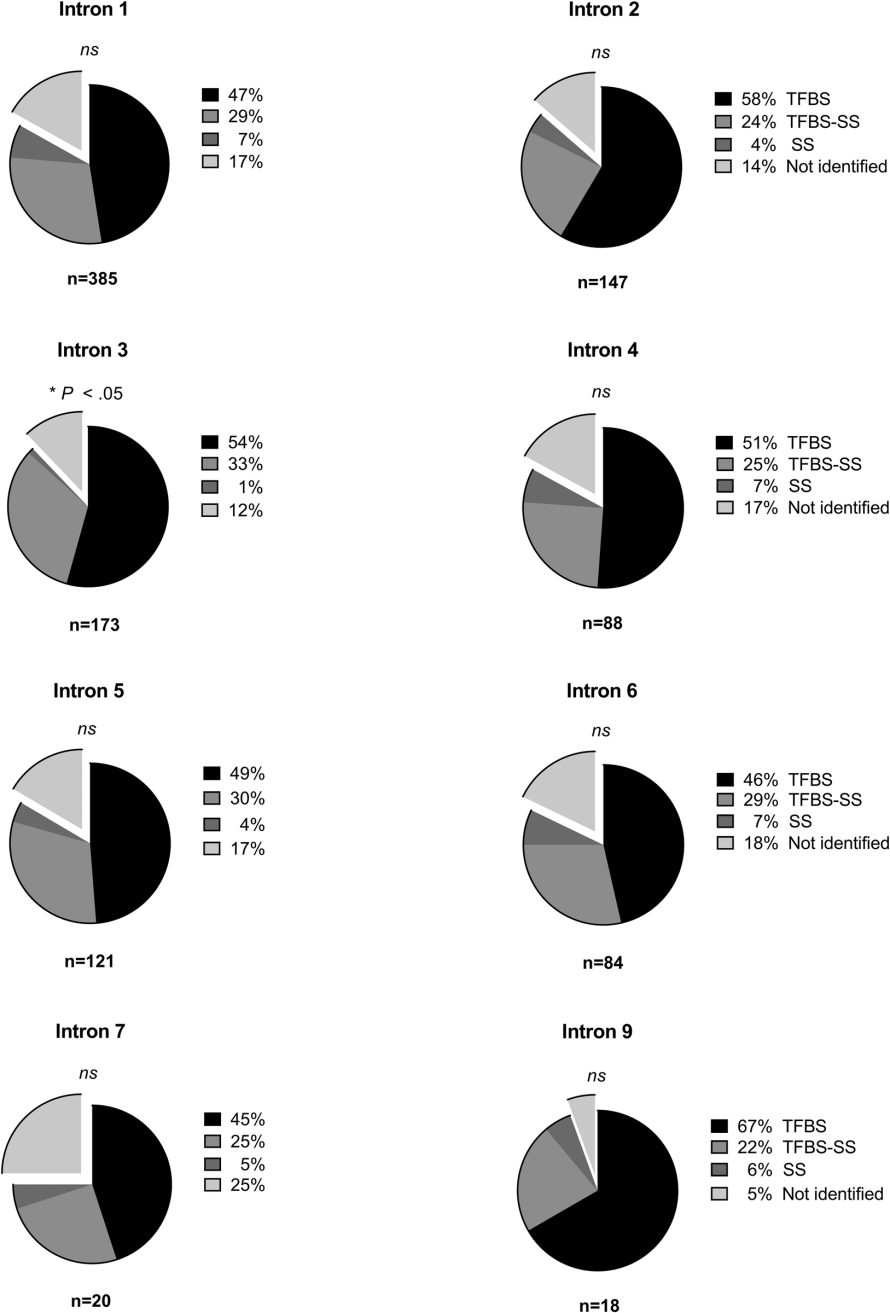
Frequency distribution of conserved pattern groups in intron 1–9 for the 25 analyzed genes. Conserved patterns were classified into four different groups: TFBS, TFBS-SS, SS, and not identified. The TFBS-SS group contained patterns that were identified as both TFBS and SS. Intron 8 is excluded due to the low number of conserved patterns shown in this intron (*n* = 8). Frequency distribution of the four groups (TFBS, TFBS-SS, SS, and Not identified) among introns was analyzed with Chi-square test (*P < 0.05, **P < 0.01, ***P < 0.001, ****P < 0.0001).

The distribution of conserved patterns for TFBS, TFBS-SS, SS, and not identified among different introns are presented in Figure 2. The TFBS group showed a similarity with 45–67% of the patterns, except for intron 8, which showed a very low number of conserved patterns (Figure S2). The sum of percentages for the groups TFBS-SS and SS was also conserved among introns with 28–36% (Figure 2). The analysis of the expected frequency distribution of the four groups showed that there were no significant differences among introns, except for intron 3 (*P* < 0.05, Figure 2) and 8 (*P* < 0.05, Figure S2).

**Figure 3:**
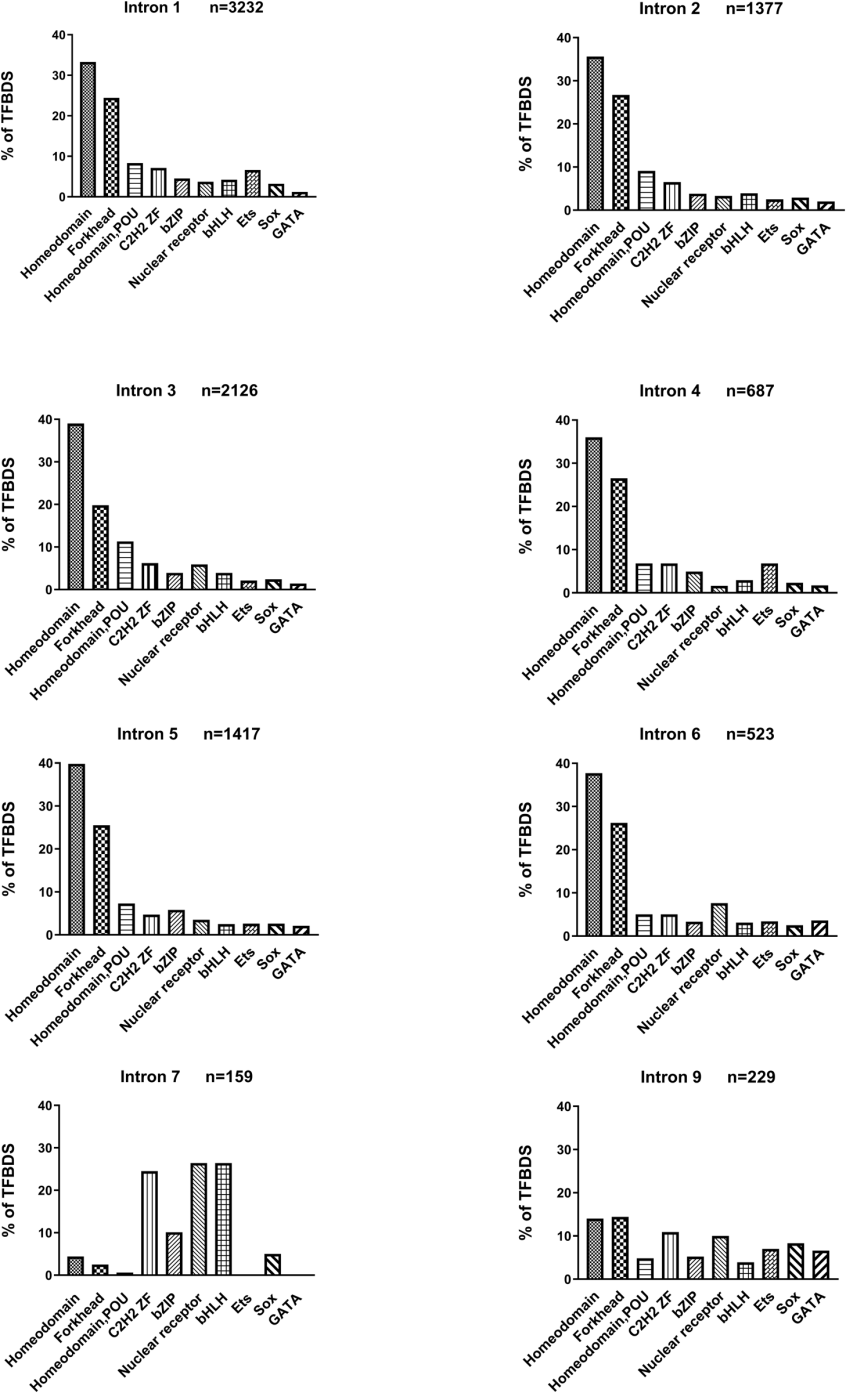
Distribution of the identified TFBDs for the analyzed TFBS, among the ten most numerous domain family types: Homeodomain, Forkhead, Homeodomain POU, C2H2 ZF, bZIP, Nuclear receptor, bHLH, Ets, Sox, and GATA. Intron 8 is excluded due to the low number of conserved patterns shown in this intron (*n* = 8).

A total of 10,608 TFBS were obtained from scanning the NRS conserved patterns in the CIS-BP database. Thirty-seven different TFBD families were identified from the TFBS analysis, of which ten with the highest number of TFBS were chosen for further analysis (Figure 3). The predominant TFBD families among the different introns were the Homeodomain, Forkhead, and Homeodomain POU domain families. Introns 7, 8, and 9 did not share the same distribution as introns 1–6 (Figure 3) (Figure S2). The same most numerous ten family domains as for the conserved sequences were obtained for the non-conserved sequences (Tables S6–S9). However, a lower abundancy of TFBDs than expected was identified in conserved sequences than in non-conserved sequences (*P* < 0.05) (Table S10).

Further analysis of the TFBDs revealed that 4.8% of the total number of conserved sequences in introns are NR-binding domains, when compared with 16.3% in exons (Tables S11 and S12). NRs bind in clusters to the same sequence but the NRs binding to the clusters differed between introns and exons (Tables S13 and S14) (Tables 2 and 3). Analyses of the three genes involved in prostate cancer etiology, *VDR* (Vitamin D receptor gene), *AR,* and *RXRA* (Retinoid X receptor alpha gene) show that there are putative binding sequences for other NRs in introns and exons (e.g., ESR, AR, PGR, RARA, RORA, RORB, RORC, RXRA, RXRB, and RXRG), suggesting crosstalk between endocrine systems (Tables 2 and 3). Furthermore, several of the TFBDs were identified as p53 transcription factor domains binding with specific TFBS in the intronic regions of five NR genes (Table S15).

**Table 2:** Nuclear receptor-binding domains detected in the intronic conserved sequences of three NR genes involved in prostate cancer etiology

Gene	Location	Nuclear receptor
VDR	Intron 3	NR2F6, NR2F1, NR2F2, HNF4A, HNF4G
AR	Intron 1	ESR1, ESR2, PGR, AR, NR2E1, RXRA, RXRB, RXRG, NR3C1, NR3C2, NR2E3, NR4A1, NR4A2, NR4A3, ESRRB
	Intron 3	NR4A1, NR4A2, NR4A3, HNF4A, HNF4G, RORA, RORB, RORC, NR2E1
	Intron 5	VDR, ESR1, ESR2, PGR, AR, HNF4A, HNF4G, NR1H4, NR1H3, NR3C1, NR3C2
	Intron 7	VDR, PGR, AR, NR3C1, NR3C2
RXRA	Intron 1	RARB, RARA, RARG

**Table 3:** Nuclear receptor-binding domains detected in the exonic conserved sequences of three NR genes involved in prostate cancer etiology

Gene	Location	Nuclear receptor
VDR	Exon 4	PPARD
	Exon 7	RORA, RORB, RORC, RARA, RARB, NR1D1, NR1D2, NR2F1 NR2F2, NR2F6, PPARA, NR2C2
AR	Exon 4	VDR, ESR1, ESR2, NR1H2, NR1H3, NR1H4, RORA, RORB, RORC, RARA, RARB, RARG, NR2C1, NR2C2, RXRA, RXRB, RXRG, NR2F1, NR2F2, NR2F6, HNF4A, HNF4G, NR2E1, PPARA, PPARD, PPARG, NR5A2, NR4A1, NR5A1, NR6A1, NR4A3, THRA, THRB, NR1D1, NR1D2
	Exon 7	ESR1, ESR2, NR1H2, NR1H3, RARB, PPARA, PPARG, PPARD, NR5A2, NR2C1, NR2C2, NR4A1, NR6A1, NR1D1, NR1D2, RARA, RARG, RORA, RORB, RORC, NR4A2, NR4A3, RXRA, RXRB, RXRG, NR2F1, NR2F2, NR2F6
RXRA	Exon 4	RORA, RORB, RORC, RARA, RARB, RARG, RXRA, RXRB, RXRG
	Exon 6	PPARA, PPARD, PPARG, NR5A1, NR5A2, NR6A1, NR2F1, NR2F2, NR2F6, NR2C1, NR2C2
	Exon 9	NR1D1, NR1H2, NR1H3, ESR1, ESR2, THRA, THRB

## 4 Discussion

The current study suggests that intronic as well as exonic sequences may be active parts in regulating gene expression, through CREs, and may serve as a target for steroid hormones. Our results show that conserved NRS intronic sequences are more abundant in the first than other introns, are enriched with TFBS, and contain SS, which are often co-localized with TFBS. Several TFBD families for TFBS were found in intron sequences, some of which contained specific TFBS for NR and p53. The intronic NR binding domains amounted to one-third of the NR binding domains found in exons.

In concordance with previous studies, the present study revealed that the number of conserved sequences decreased with intron position, indicating that intronic conserved pattern density among several species is higher in the first intron than introns downstream [10,36,37]. However, the density of conserved patterns was higher in the present study when compared with the study by Park et al. [10]. This is perhaps due to the number and evolutionary span of species analyzed in each study (five and 46 mammalian species, respectively). Furthermore, Park et al. excluded sequences within 300 base pairs of the splice junction (the boundary between intron and exon) in their analysis [10]. The current study included such sequences since they may contain regulatory elements, such as TFBS and SS. These regulatory elements could play a role in the transcription and/or splicing process and be responsible for the expression of certain genetic splicing variants [38,39]. In contrast to the results by Park et al., where a low number of conserved patterns were found downstream of the second intron [10], the results in the current study identified high frequencies of conserved patterns downstream of the first intron (Table S5). Thus, taken together, the current study suggests that most of the introns studied may include conserved patterns containing CREs, further suggesting a role in regulating gene transcription.

Consistent with the previous report [10], the present study shows that putative TFBS are more abundant within the first introns, with the highest abundance in the first intron. The proportion of TFBS relative to the conserved sequences is preserved among introns, which has not been previously reported. This suggests that the number of TFBS is directly related to the number of conserved sequences and may explain the low number of TFBS obtained in the last introns. According to the present study’s analysis of the false-positive ratio, due to sequence overlap, a considerable number of TFBS were bioinformatically confirmed to be true putative TFBS. However, further experimental validations are needed, such as ChIP-on-chip or luciferase assays [40,41]. Several studies agree that the identification of TFBS is complex and usually results in a large number of both false positives and false negatives [40,41,42]. These studies reduced the false-positive ratio by scanning the genome with PWM and identified true TFBS by searching for conservation of these sequences in orthologous transcripts. A conclusion from these studies is that TFBS can be identified through studying sequence conservation alone [40,41,42]. However, not all the functional TFBS present in the genome or sequences analyzed may be identified by conservation [40,41,42]. The results in the present study are in line with previous observations [40,41,42] showing that there is a higher percentage of TFBS within conserved regions compared to non-conserved regions.

To our knowledge, this is the first study to report an equal distribution of the conserved sequence types (TFBS and SS) among introns. The underlying cause of this preservation of distribution among introns is so far unknown but may be related to the regulation of the expression of certain genes and/or their splicing. Interestingly, the present study found that the proportion of SS in conserved sequences was greater when co-localized with TFBS (TFBS-SS group) than alone. Based on this finding, we propose that specific conserved sequences from NRS can act as splice consensus sequences and that most of them are surrounded by TFBS sites. Previous studies have suggested multiple links between transcription and splicing and that there are difficulties to isolate both processes since they are closely connected [43,44]. Furthermore, crosstalk between proteins involved in both transcription and pre-mRNA splicing has been suggested, and several mammalian candidate proteins, including transcription factors, have been identified [43,45]. Thus, we suggest that TFBS closely located to SS, may act as transcription factors or splicing binding sequences, and support previous studies suggesting a link between the transcriptional and the spliceosomal complex.

Mapping of the functional domains of transcription factors is crucial to understand their molecular function [46]. In the present study, the same ten TFBD families were more numerous than other families in both non-conserved sequences and conserved sequences (Figure S2), which suggests that these ten families are common TFBD families for intronic sequences of NRs. However, there is less consensus between introns regarding TFBD family enrichment in non-conserved sequences. Considering this, conserved sequences may be more similar in their nucleotide composition and thus bind with specific TFBD families.

Further analyses of the putative TFBS identified in this study revealed specific TFBS for p53 in intronic conserved sequences of five NR genes: The nuclear receptor subfamily 2 group F (*NR2F)*, the estrogen receptor 1 (*ESR1)*, the nuclear receptor subfamily 4 Group A member 3 (*NR4A3)*, the *AR* and the nuclear receptor subfamily 1 group D member 1 (*NR1D1)*. A previous study mapping p53 binding sites in the whole genome did not find p53 specific TFBS for NR intronic sequences [47]. Possible explanations for these different findings could be that Wei et al. conducted their study on cultured cancer cells, *in vitro*, and that their analysis was performed on expression level but not on a single molecular level [47]. Furthermore, mapping was done for the whole genome and only detected the regions highly enriched with p53-binding sites [47]. On the other hand, the present study was performed at normal conditions and not under conditions influenced by transcriptional rates, where eight p53 TFBS specific for the NRs was identified. Thus, the current finding extends the knowledge about p53 binding locations and indicates that p53 is involved in regulating NR-mediated transcription.

This is to our knowledge the first study to report putative interactions of steroid NRs in the case of intronic sequences. These results indicate the existence of a regulatory network involving the interaction of regulatory DNA elements located in the intronic regions of the NR genes and NR transcription factors. Of the three steroid NR genes studied, NR binding domains were demonstrated in introns 1 and 3 and exons 4, 6, 7, and 9, and thus may indicate crosstalk between endocrine systems. Crosstalk has been described for steroid NRs [25], growth factor receptors [48], steroids [49], intracellular and stress-activated kinases within the mitogen-activated protein kinase (MAPK) superfamily [50,51] as well as downstream signaling components of these kinase pathways [52,53]. Understanding the crosstalk between steroid receptors is important in the initiation and progression of hormone-driven cancers [21,25,54]. In the present study, *VDR*, *AR,* and *RXRA* have several TFBS for NRs in introns and exons, which are closely connected to cancer development. The current results suggest a crosstalk between the androgen endocrine system and the VDR, the ESR, the progesterone receptor (PGR), and the retinoic acid receptors as well as a crosstalk between the retinoic acid endocrine system and ESR and the thyroid hormone receptor (THR), among others. These findings are in concert with previous reports on the crosstalk between steroid receptors of prostate and breast cancer cells [21,25]. It is known that the protein interaction between VDR and RXR causes antitumoral effects in prostate cancer [55,56]. Further examples are ARs, which either have an antagonistic or a cooperative effect on the ESR binding to estradiol responsive elements dependent on the presence of dihydrotestosterone [57,58,59]. Thus, the present study provides more evidence for crosstalk between steroid NRs and contributes with novel targets for steroid NR regulation. Furthermore, this study confirms the hypothesis that individual steroids and steroid NRs rarely work in isolation but rather as a crosstalk between different receptor types, allowing activation of signaling pathways, and modulate transcriptional responses [25].

## Abbreviations

AR: androgen receptor
BCSDm: basic conserved sequence detection method
*BRCA1*: breast cancer type 1 susceptibility protein
*BRCA2*: breast cancer type 2 susceptibility protein
CNS: conserved non-coding sequence
CRE: *cis*-regulatory element
ERα: estrogen receptor α
ESR1: estrogen receptor 1
HSF: human splicing finder
MAPK: mitogen-activated protein kinase
NR: nuclear receptor
NR4A3: nuclear receptor subfamily 4 group A member 3
NR1D1: nuclear receptor subfamily 1 group D member 1
NR2F: nuclear receptor subfamily 2 group F
NRS: nuclear receptor superfamily
PGR: progesterone receptor
PSSM: position score matrix
PWM: position weight matrix
RXRA: retinoid X receptor-alpha
THR: thyroid hormone receptor
TFBD: transcription factor binding domain
TFBS: transcription factor binding sites
SS: splicing sites
SFBS: splicing factor binding sites
VDR: vitamin D receptor
